# Common Variants Related to Serum Uric Acid Concentrations Are Associated with Glucose Metabolism and Insulin Secretion in a Chinese Population

**DOI:** 10.1371/journal.pone.0116714

**Published:** 2015-01-24

**Authors:** Xue Sun, Rong Zhang, Feng Jiang, Shanshan Tang, Miao Chen, Danfeng Peng, Jing Yan, Tao Wang, Shiyun Wang, Yuqian Bao, Cheng Hu, Weiping Jia

**Affiliations:** 1 Shanghai Diabetes Institute, Shanghai Clinical Center for Diabetes, Shanghai Key Clinical Center for Metabolic Disease, Shanghai Key Laboratory of Diabetes Mellitus, Shanghai Jiao Tong University Affiliated Sixth People’s Hospital, Shanghai, 200233, China; 2 Shanghai Jiao Tong University Affiliated Sixth People’s Hospital South Campus, Shanghai, 201400, China; MOE Key Laboratory of Environment and Health, School of Public Health, Tongji Medical College, Huazhong University of Science and Technology, CHINA

## Abstract

**Background:**

Elevated serum uric acid concentration is an independent risk factor and predictor of type 2 diabetes (T2D). Whether the uric acid-associated genes have an impact on T2D remains unclear. We aimed to investigate the effects of the uric acid-associated genes on the risk of T2D as well as glucose metabolism and insulin secretion.

**Method:**

We recruited 2,199 normal glucose tolerance subjects from the Shanghai Diabetes Study I and II and 2,999 T2D patients from the inpatient database of Shanghai Diabetes Institute. Fifteen single nucleotide polymorphisms (SNPs) mapped in or near 11 loci (*PDZK1, GCKR, LRP2, SLC2A9, ABCG2, LRRC16A, SLC17A1, SLC17A3, SLC22A11, SLC22A12* and *SF1*) were genotyped and serum biochemical parameters related to uric acid and T2D were determined.

**Results:**

*SF1* rs606458 showed strong association to T2D in both males and females (*p* = 0.034 and 0.0008). In the males, *LRRC16A* was associated with 2-h insulin and insulin secretion (*p* = 0.009 and 0.009). *SLC22A11* was correlated with HOMA-B and insulin secretion (*p* = 0.048 and 0.029). *SLC2A9* rs3775948 was associated with 2-h glucose (*p* = 0.043). In the females, *LRP2* rs2544390 and rs1333049 showed correlations with fasting insulin, HOMA-IR and insulin secretion (*p* = 0.028, 0.033 and 0.052 and *p* = 0.034, 0.047 and 0.038, respectively). *SLC2A9* rs11722228 was correlated with 2-h glucose, 2-h insulin and insulin secretion (*p* = 0.024, 0.049 and 0.049, respectively).

**Conclusions:**

Our results indicated that the uric acid-associated genes have an impact on the risk of T2D, glucose metabolism and insulin secretion in a Chinese population.

## Introduction

The prevalence of type 2 diabetes (T2D) is increasing exponentially worldwide, promoted by multifactorial genetic or environmental factors. T2D is characterised by chronic hyperglycemia caused by insulin resistance and relative insulin deficiency [1]. Recent evidence has emerged from several large epidemiological studies indicating that serum uric acid levels are an independent risk factor and predictor of T2D, with a 17% increment in the risk of T2D per 1 mg/dl increase in serum uric acid levels [2]. Uric acid levels are positively associated with fasting plasma glucose [3], impaired fasting glucose [4] and 2-hour postload glucose [5]. In addition, insulin resistance and impaired insulin secretion are strongly influenced by uric acid levels. Cross-sectional data from 8,144 individuals in Japan found that uric acid levels were significantly correlated with HOMA-IR [6]. In rats, the insulin resistance results of fructose-induced elevated uric acid levels could be improved using xanthine oxidase inhibitors or uricosuric agents [7]. Uric acid could cause oxidative damage and function inhibition in pancreatic β-cells through many signalling pathways, including adenosine monophosphate-activated protein kinase (AMPK), extracellular signal-regulated kinase (ERK) [8] and nuclear factor-κB (NF-κB) [9]. Additionally, glucose-induced insulin secretion was inhibited by uric acid, which further elevated serum glucose levels. In the residual β-cells in T2D patients, uric acid enhanced the ability of insulin secretion to compensate for the lack of insulin action [10, 11]. In accordance with this phenomenon, it was observed that uric acid linearly increased with increasing serum insulin levels in newly diagnosed diabetic patients. Concurrently, the enhanced residual β-cell function appears to decay more rapidly [11]. As polygenetic diseases, serum uric acid level disorders and T2D are determined by genetic factors, with up to 42% [12] and 10% heritability, respectively. Recently, the *SLC2A9* gene, which encodes the solute carrier family 2 (which could serve as glucose transporter 9 and facilitate the transport of glucose, fructose, and uric acid), has been associated with T2D [13]. Additionally, the T2D-associated gene, *GCKR*, has been reported to be involved in regulating serum uric acid levels [14]. The interaction between uric acid-associated and T2D-associated genes is important for investigating the relationship of serum uric acid levels and the risk of T2D, glucose metabolism and insulin secretion. In this study, we selected 2,999 T2D patients and 2,199 normal glucose tolerance (NGT) subjects to explore whether uric acid-associated genes affect the T2D risk, glucose metabolism and insulin secretion.

## Methods

### Ethics statement

This study was approved by the Institutional Review Board of Shanghai Jiao Tong University Affiliated Sixth People’s Hospital, in accordance with the principles of the Helsinki Declaration II. Written informed consent was obtained from each participant.

### Participants

We recruited 5,198 participants, including 2,199 NGT subjects from the Shanghai Diabetes Study I and II [15, 16]and 2,999 T2D patients from the inpatient database of Shanghai Diabetes Institute predominantly with the identical genetic background (i.e., eastern Han Chinese ancestry) and residing in Shanghai or nearby regions. The NGT subjects had a negative family history of diabetes and were assessed with standard 75-g oral glucose tolerance tests (OGTTs). We excluded the participants with cancer, hepatic disease, renal disease or other comorbidities.

### Clinical measurements

The phenotypes of the anthropometric and biochemical traits related to uric acid, glucose metabolism and insulin secretion levels were extensively evaluated in all participants. Height (m) and weight (kg) were measured, and body mass index (BMI) was calculated as weight/height^2^. OGTTs were performed in the morning after an overnight fast. Blood samples were obtained at the fasting and at 2 h during the OGTTs. The plasma glucose and serum insulin were measured. Basal insulin sensitivity and beta cell function were calculated from the fasting plasma glucose and insulin using HOMA [17]. First- and second-phase insulin secretions were estimated using the glucose and insulin levels at 0 and 120 min during the OGTT and BMI measurements [18].

### SNP selection, genotyping and quality control analysis

Fifteen SNPs from or near 11 loci (*PDZK1* rs12129861, *GCKR* rs780094, *LRP2* rs2544390, *SLC2A9* rs11722228, rs16890979, rs3775948 and rs10489070, *ABCG2* rs2231142, *LRRC16A* rs742132, *SLC17A1* rs1183201, *SLC17A3* rs1165205, rs1333049, *SLC22A11* rs17300741, *SLC22A12* rs506338, *SF1* rs606458) were selected based on the literature and have been reported to be associated with serum uric acid levels [14, 19]. The SNPs were genotyped using a multiplex primer extension, with detection by matrix-assisted laser desorption/ionisation time-of-flight mass spectroscopy with a MassARRAY Compact Analyzer (Sequenom, San Diego, CA, USA). All 15 SNPs passed the quality control criteria with genotyping call rates greater than 90%. Individuals with more than 10% of the genotypes missing were excluded.

### Statistical analysis

The continuous variables were expressed as the mean ± standard deviation or median (interquartile range) and were compared between the T2D and NGT study participants using Student’s t test. Before the association analysis, the T2D patients and NGT subjects performed the Hardy-Weinberg equilibrium test. We excluded the SNPs that failed the Hardy-Weinberg equilibrium test (*p*<0.01). Tests of normality were conducted for all quantitative traits. The allelic frequencies of the diabetic patients and controls were compared using a χ^2^-test, and the ORs with 95% CIs are presented. The quantitative traits were analysed by linear regression using the additive model, with adjustments for age, gender and BMI using PLINK [20], and the regression coefficients ± standard error were presented, with 95% CIs. The skewed distributed quantitative traits, including the fasting and 2-h insulin levels, HOMA-B, HOMA-IR and STUMVOLL, were logarithmically transformed (log_10_) to approximate the univariate normality. To adjust for multiple comparisons, 10,000 permutations (using PLINK) were performed for each trait to assess the empirical p values. Statistical analyses were performed using SAS for Windows (version 8.0; SAS Institute, Cary, NC, USA), unless otherwise specified. A two-tailed *p* value of *<* 0.05 was considered to be statistically significant.

## Results

The clinical characteristics of the participants are shown in Table 1. The genotype frequencies of all polymorphisms were in Hardy-Weinberg equilibrium. Table 2 shows the analyses of the associations between these SNPs and type 2 diabetes in the males, females and all participants. *GCKR* rs780094 and *SF1* rs606458 demonstrated associations with T2D in the females (adjusted for age and BMI, OR = 1.276, 95% CI, 1.114–1.463, *p* = 0.0004; OR = 1.269, 95% CI, 1.105–1.458, *p* = 0.0008) and in all participants (adjusted for age, BMI and sex, OR = 1.223, 95% CI, 1.111–1.346, *p* = 3.94E-05; OR = 1.107, 95% CI, 1.005–1.220, *p* = 0.038). Only rs606458 was associated with T2D in the males, after being adjusted for age and BMI (OR = 1.162, 95% CI, 1.011–1.334, *p* = 0.034). However, after correcting multiple comparisons with 10,000 permutations, the effects of *GCKR* rs780094 and *SF1* rs606458 in the females (empirical *p* = 0.005 and 0.009) and *GCKR* rs780094 in all participants (empirical *p* = 0.0004) on T2D remained significant.

**Table 1:** Clinical characteristics of the study samples.

	NGT	T2D
Samples	2199	2999
Male/female(n)	824(1375)	1556(1443)
Age (years)	50(39, 59)	61(52, 71)
Body mass index (kg/m^2^)	23.086(21.171, 25.229)	24.000(21.500, 26.420)
Systolic blood pressure (mm Hg)	120(110, 130)	130(120, 150)
Diastolic blood pressure (mm Hg)	80(70, 80)	80(75, 90)
Blood urea nitrogen (mmol/L)	4.600(3.900, 5.400)	5.600(4.600, 6.900)
Serum creatinine (umol/L)	63(54, 75)	67(56, 82)
Uric acid (umol/L)	282(235, 341)	306(251, 370)
Total cholesterol (mmol/L)	4.560(3.940, 5.200)	4.600(4.000, 5.400)
Triglycerides (mmol/L)	1.120(0.790, 1.630)	1.460(0.960, 2.150)
High-density lipoprotein cholesterol(mmol/L)	1.350(1.140, 1.560)	1.100(0.920, 1.320)
Low-density lipoprotein. cholesterol(mmol/L)	2.840(2.350, 3.390)	2.930(2.350, 3.530)
Fasting glucose (mmol/L)	5.180(4.800, 5.500)	-
2-h glucose (mmol/L)	5.550(4.710, 6.400)	-
Fasting Insulin (mU/L)	5.620(4.110, 7.750)	-
2-h insulin (mU/L)	19.235(11.010, 34.140)	-
Homa-IR	1.207(0.874, 1.709)	-
Homa-B	82.007(54.489, 126.901)	-
Stumvoll-First phase insulin secretion (pmol/L)	134.216(76.685, 238.029)	-
Stumvoll-second phase insulin secretion (pmol/L)	256.166(218.691, 294.651)	-

Data are shown as median (interquartile range) or n. NGT: normal glucose tolerance. T2D: type 2 diabetes.

**Table 2:** Effects of SNPs from eleven uric acid associated loci on type 2 diabetes susceptibility in the Chinese population.

Loci	SNP	Chr*	Position(bp)	Effect/Other Allele	MAF	**Males**			**Females**			**ALL**		
						OR	95% CI	*P* value	OR	[95% CI]	*P* value	OR	95% CI	*P* value
*PDZK1*	rs12129861	1	145725689	A/G	0.189	0.970	0.822; 1.145	0.722	1.081	0.914; 1.279	0.365	1.032	0.919; 1.160	0.592
*GCKR*	rs780094	2	27741237	G/A	0.451	1.162	1.011; 1.334	**0.034**	1.276	1.114; 1.463	**0.0004**	1.223	1.111; 1.346	**3.94E-05**
*LRP2*	rs2544390	2	170204846	T/C	0.456	0.977	0.856; 1.115	0.729	0.959	0.842; 1.092	0.527	0.969	0.884; 1.062	0.501
*SLC2A9*	rs11722228	4	9915741	T/C	0.293	0.939	0.814; 1.083	0.386	1.122	0.968; 1.300	0.127	1.011	0.914; 1.119	0.831
*SLC2A9*	rs16890979	4	9922167	T/C	0.015	0.981	0.542; 1.773	0.949	1.059	0.618; 1.814	0.835	0.978	0.662; 1.445	0.911
*SLC2A9*	rs3775948	4	9995182	C/G	0.410	1.075	0.940; 1.228	0.291	0.966	0.844; 1.104	0.610	1.026	0.934; 1.126	0.591
*SLC2A9-WDR1*	rs10489070	4	10276352	C/G	0.138	0.925	0.765; 1.118	0.418	0.994	0.820; 1.206	0.954	0.959	0.839; 1.096	0.539
*ABCG2*	rs2231142	4	89052323	A/C	0.312	0.954	0.827; 1.101	0.519	0.917	0.793; 1.062	0.247	0.937	0.847; 1.037	0.210
*LRRC16A*	rs742132	6	25607571	C/T	0.257	0.927	0.802; 1.072	0.308	1.074	0.921; 1.252	0.363	1.014	0.913; 1.126	0.798
*SLC17A1*	rs1183201	6	25823444	T/A	0.157	0.940	0.788; 1.122	0.496	0.996	0.832; 1.193	0.968	0.980	0.864; 1.110	0.746
*SLC17A3*	rs1165205	6	25870542	A/T	0.160	0.932	0.783; 1.109	0.426	1.009	0.845; 1.206	0.918	0.980	0.866; 1.109	0.748
	rs1333049	9	22125503	C/G	0.451	0.983	0.862; 1.121	0.796	1.054	0.923; 1.204	0.435	1.024	0.934; 1.124	0.609
*SLC22A11*	rs17300741	11	64331462	G/A	0.057	0.992	0.745; 1.321	0.956	0.956	0.720; 1.276	0.772	0.972	0.795; 1.187	0.777
*SLC22A12*	rs506338	11	64440920	C/T	0.261	1.024	0.882; 1.189	0.752	0.990	0.852; 1.149	0.893	0.991	0.893; 1.100	0.867
*SF1*	rs606458	11	64546391	G/A	0.354	0.985	0.858; 1.131	0.829	1.269	1.105; 1.458	**0.0008**	1.107	1.005; 1.220	**0.038**

*Chromosome.

*P* values were adjusted for age, gender and BMI in all participants and were adjusted for age and BMI in both female and males. *P* values < 0.05 were shown in bold. Position is given for GRCh37.p13. The effect allele is the allele to which the β estimate refers.

We then analysed the effects of these SNPs on the quantitative traits in the males and females with NGT. In the males, *LRRC16A* rs742132 was significantly associated with the serum 2-h insulin and insulin secretion indices Stumvoll first phase insulin secretion and marginally associated with second phase (*p* = 0.009, 0.009 and 0.058; empirical *p* = 0.131, 0.126 and 0.572). *SLC22A11* rs17300741 was correlated with HOMA-B and Stumvoll second phase insulin secretion (*p* = 0.048 and 0.029; empirical *p* = 0.495 and 0.348). *SLC22A12* rs506338 showed significant correlation with HOAM-B (*p* = 0.030; empirical *p* = 0.347). *SLC2A9* rs3775948 was shown to be associated with 2-h glucose (*p* = 0.043; empirical *p* = 0.470) (Table 3 and Table 4).

**Table 3:** Association between SNPs from fifteen loci and glucose and insulin levels in males with normal glucose regulation.

Loci	SNP	Effect/Other Allele	Fasting glucose (n = 824)		2-h glucose (n = 824)		Fasting insulin (n = 455)		2-h insulin (n = 455)	
*PDZK1*	rs12129861	A/G	Beta	-0.013±0.032	Beta	-0.053±0.070	Beta	-0.008±0.022	Beta	-0.021±0.031
			95% CI	[-0.075;0.049]	95% CI	[-0.191;0.084]	95% CI	[-0.051;0.036]	95% CI	[-0.082;0.040]
			*P* value	0.676	*P* value	0.447	*P* value	0.729	*P* value	0.503
*GCKR*	rs780094	G/A	Beta	0.047±0.026	Beta	-0.033±0.059	Beta	-0.001±0.020	Beta	-0.020±0.028
			95% CI	[-0.004;0.098]	95% CI	[-0.148;0.082]	95% CI	[-0.039;0.038]	95% CI	[-0.074;0.033]
			*P* value	0.069	*P* value	0.573	*P* value	0.968	*P* value	0.458
*LRP2*	rs2544390	T/C	Beta	-0.004±0.025	Beta	0.013±0.056	Beta	-0.021±0.019	Beta	-0.004±0.027
			95% CI	[-0.053;0.045]	95% CI	[-0.097;0.123]	95% CI	[-0.058;0.017]	95% CI	[-0.057;0.049]
			*P* value	0.875	*P* value	0.815	*P* value	0.275	*P* value	0.879
*SLC2A9*	rs11722228	T/C	Beta	-0.043±0.027	Beta	0.047±0.060	Beta	0.030±0.020	Beta	0.047±0.028
			95% CI	[-0.096;0.009]	95% CI	[-0.070;0.164]	95% CI	[-0.010;0.069]	95% CI	[-0.009;0.103]
			*P* value	0.108	*P* value	0.427	*P* value	0.141	*P* value	0.101
*SLC2A9*	rs16890979	T/C	Beta	0.105±0.114	Beta	-0.205±0.255	Beta	0.059±0.105	Beta	0.034±0.149
			95% CI	[-0.119;0.329]	95% CI	[-0.705;0.296]	95% CI	[-0.147;0.266]	95% CI	[-0.258;0.327]
			*P* value	0.360	*P* value	0.424	*P* value	0.574	*P* value	0.818
*SLC2A9*	rs3775948	C/G	Beta	0.019±0.025	Beta	-0.114±0.056	Beta	-0.016±0.019	Beta	-0.013±0.027
			95% CI	[-0.031;0.068]	95% CI	[-0.225;-0.004]	95% CI	[-0.054;0.022]	95% CI	[-0.067;0.040]
			*P* value	0.462	*P* value	**0.043**	*P* value	0.406	*P* value	0.625
*SLC2A9-WDR1*	rs10489070	C/G	Beta	0.053±0.036	Beta	-0.003±0.080	Beta	-0.028±0.027	Beta	-0.059±0.038
			95% CI	[-0.017;0.123]	95% CI	[-0.159;0.154]	95% CI	[-0.081;0.026]	95% CI	[-0.133;0.016]
			*P* value	0.138	*P* value	0.972	*P* value	0.309	*P* value	0.123
*ABCG2*	rs2231142	A/C	Beta	-0.021±0.027	Beta	0.036±0.060	Beta	0.001±0.020	Beta	0.0004±0.029
			95% CI	[-0.074;0.032]	95% CI	[-0.082;0.154]	95% CI	[-0.039;0.041]	95% CI	[-0.057;0.056]
			*P* value	0.435	*P* value	0.550	*P* value	0.955	*P* value	0.990
*LRRC16A*	rs742132	C/T	Beta	-0.015±0.027	Beta	-0.073±0.060	Beta	-0.031±0.020	Beta	-0.073±0.028
			95% CI	[-0.068;0.038]	95% CI	[-0.190;0.044]	95% CI	[-0.070;0.008]	95% CI	[-0.127;-0.018]
			*P* value	0.581	*P* value	0.224	*P* value	0.122	*P* value	**0.009**
*SLC17A1*	rs1183201	T/A	Beta	0.041±0.033	Beta	0.011±0.073	Beta	0.005±0.025	Beta	0.035±0.035
			95% CI	[-0.023;0.106]	95% CI	[-0.133;0.155]	95% CI	[-0.043;0.053]	95% CI	[-0.033;0.102]
			*P* value	0.212	*P* value	0.882	*P* value	0.840	*P* value	0.317
*SLC17A3*	rs1165205	A/T	Beta	0.051±0.032	Beta	-0.009±0.072	Beta	0.003±0.024	Beta	0.023±0.034
			95% CI	[-0.012;0.114]	95% CI	[-0.151;0.133]	95% CI	[-0.044;0.051]	95% CI	[-0.045;0.090]
			*P* value	0.116	*P* value	0.900	*P* value	0.886	*P* value	0.511
	rs1333049	C/G	Beta	0.025±0.025	Beta	0.039±0.056	Beta	-0.007±0.019	Beta	-0.014±0.026
			95% CI	[-0.025;0.074]	95% CI	[-0.070;0.149]	95% CI	[-0.044;0.029]	95% CI	[-0.066;0.037]
			*P* value	0.327	*P* value	0.480	*P* value	0.696	*P* value	0.584
*SLC22A11*	rs17300741	G/A	Beta	-0.049±0.055	Beta	-0.196±0.123	Beta	0.059±0.042	Beta	0.055±0.059
			95% CI	[-0.157;0.059]	95% CI	[-0.436;0.0450]	95% CI	[-0.023;0.141]	95% CI	[-0.061;0.171]
			*P* value	0.376	*P* value	0.111	*P* value	0.158	*P* value	0.354
*SLC22A12*	rs506338	C/T	Beta	-0.029±0.029	Beta	-0.042±0.064	Beta	0.031±0.022	Beta	-0.012±0.031
			95% CI	[-0.085;0.027]	95% CI	[-0.166;0.083]	95% CI	[-0.012;0.073]	95% CI	[-0.072;0.048]
			*P* value	0.314	*P* value	0.514	*P* value	0.156	*P* value	0.700
*SF1*	rs606458	G/A	Beta	-0.023±0.027	Beta	-0.004±0.059	Beta	0.002±0.020	Beta	-0.027±0.028
			95% CI	[-0.075;0.029]	95% CI	[-0.120;0.112]	95% CI	[-0.037;0.041]	95% CI	[-0.082;0.028]
			*P* value	0.395	*P* value	0.950	*P* value	0.914	*P* value	0.332

*P* values were adjusted for age and BMI. *P* values < 0.05 were shown in bold. Log transformed (log_10_) values were used for fasting and 2-h insulin levels. The effect allele is the allele to which the β estimate refers.

**Table 4:** Association between SNPs from fifteen loci and insulin secretion and sensitivity indices in males with normal glucose regulation.

Loci	SNP	Effect/Other Allele	HOMA-IR (n = 455)		HOMA-B (n = 455)		Stumvoll-First phase (n = 453)		Stumvoll-second phase (n = 453)	
*PDZK1*	rs12129861	A/G	Beta	-0.009±0.023	Beta	-0.006±0.026	Beta	-0.021±0.031	Beta	0.005±0.009
			95% CI	[-0.054;0.035]	95% CI	[-0.056;0.045]	95% CI	[-0.082;0.041]	95% CI	[-0.012;0.022]
			*P* value	0.685	*P* value	0.830	*P* value	0.505	*P* value	0.586
*GCKR*	rs780094	G/A	Beta	0.002±0.020	Beta	-0.013±0.023	Beta	-0.021±0.028	Beta	-0.004±0.008
			95% CI	[-0.038;0.041]	95% CI	[-0.058;0.032]	95% CI	[-0.075;0.034]	95% CI	[-0.019;0.011]
			*P* value	0.934	*P* value	0.574	*P* value	0.458	*P* value	0.622
*LRP2*	rs2544390	T/C	Beta	-0.017±0.020	Beta	-0.038±0.022	Beta	-0.004±0.027	Beta	-0.003±0.007
			95% CI	[-0.055;0.021]	95% CI	[-0.082;0.006]	95% CI	[-0.057;0.049]	95% CI	[-0.017;0.012]
			*P* value	0.387	*P* value	0.088	*P* value	0.879	*P* value	0.709
*SLC2A9*	rs11722228	T/C	Beta	0.029±0.021	Beta	0.032±0.024	Beta	0.047±0.029	Beta	0.0130±0.008
			95% CI	[-0.011;0.069]	95% CI	[-0.014;0.078]	95% CI	[-0.009;0.103]	95% CI	[-0.003;0.028]
			*P* value	0.159	*P* value	0.176	*P* value	0.100	*P* value	0.108
*SLC2A9*	rs16890979	T/C	Beta	0.065±0.108	Beta	0.024±0.123	Beta	0.034±0.149	Beta	-0.012±0.041
			95% CI	[-0.146;0.276]	95% CI	[-0.217;0.265]	95% CI	[-0.258;0.327]	95% CI	[-0.092;0.069]
			*P* value	0.548	*P* value	0.845	*P* value	0.818	*P* value	0.774
*SLC2A9*	rs3775948	C/G	Beta	-0.018±0.02	Beta	-0.011±0.023	Beta	-0.014±0.028	Beta	-0.001±0.008
			95% CI	[-0.057;0.021]	95% CI	[-0.055;0.034]	95% CI	[-0.068;0.040]	95% CI	[-0.015;0.014]
			*P* value	0.364	*P* value	0.641	*P* value	0.624	*P* value	0.937
*SLC2A9-WDR1*	rs10489070	C/G	Beta	-0.020±0.028	Beta	-0.061±0.032	Beta	-0.06±0.038	Beta	-0.018±0.011
			95% CI	[-0.074;0.034]	95% CI	[-0.123;0.001]	95% CI	[-0.135;0.015]	95% CI	[-0.039;0.002]
			*P* value	0.469	*P* value	**0.055**	*P* value	0.120	*P* value	0.081
*ABCG2*	rs2231142	A/C	Beta	-0.002±0.021	Beta	0.014±0.024	Beta	0.0002±0.029	Beta	-0.001±0.008
			95% CI	[-0.043;0.039]	95% CI	[-0.033;0.060]	95% CI	[-0.057;0.057]	95% CI	[-0.016;0.015]
			*P* value	0.927	*P* value	0.568	*P* value	0.994	*P* value	0.917
*LRRC16A*	rs742132	C/T	Beta	-0.032±0.020	Beta	-0.021±0.023	Beta	-0.073±0.028	Beta	-0.015±0.008
			95% CI	[-0.071;0.008]	95% CI	[-0.066;0.024]	95% CI	[-0.128;-0.018]	95% CI	[-0.030;0.0004]
			*P* value	0.118	*P* value	0.366	*P* value	**0.009**	*P* value	**0.058**
*SLC17A1*	rs1183201	T/A	Beta	0.010±0.025	Beta	-0.009±0.029	Beta	0.035±0.035	Beta	0.010±0.010
			95% CI	[-0.039;0.059]	95% CI	[-0.065;0.047]	95% CI	[-0.033;0.103]	95% CI	[-0.009;0.028]
			*P* value	0.696	*P* value	0.763	*P* value	0.316	*P* value	0.308
*SLC17A3*	rs1165205	A/T	Beta	0.009±0.025	Beta	-0.012±0.028	Beta	0.023±0.034	Beta	0.011±0.009
			95% CI	[-0.039;0.058]	95% CI	[-0.068;0.043]	95% CI	[-0.045;0.090]	95% CI	[-0.008;0.029]
			*P* value	0.715	*P* value	0.662	*P* value	0.510	*P* value	0.245
rs1333049	C/G	Beta	-0.004±0.019	Beta	-0.017±0.022	Beta	-0.015±0.027	Beta	-0.011±0.007
			95% CI	[-0.042;0.033]	95% CI	[-0.060;0.026]	95% CI	[-0.067;0.037]	95% CI	[-0.025;0.003]
			*P* value	0.818	*P* value	0.451	*P* value	0.579	*P* value	0.124
*SLC22A11*	rs17300741	G/A	Beta	0.049±0.043	Beta	0.097±0.049	Beta	0.055±0.059	Beta	0.036±0.016
			95% CI	[-0.035;0.133]	95% CI	[0.001;0.193]	95% CI	[-0.061;0.172]	95% CI	[0.004;0.068]
			*P* value	0.253	*P* value	**0.048**	*P* value	0.354	*P* value	**0.029**
*SLC22A12*	rs506338	C/T	Beta	0.025±0.022	Beta	0.055±0.025	Beta	-0.012±0.031	Beta	0.0002±0.008
			95% CI	[-0.018;0.069]	95% CI	[0.006;0.105]	95% CI	[-0.072;0.049]	95% CI	[-0.017;0.016]
			*P* value	0.252	*P* value	**0.030**	*P* value	0.703	*P* value	0.982
*SF1*	rs606458	G/A	Beta	-0.001±0.020	Beta	0.017±0.023	Beta	-0.027±0.028	Beta	-0.006±0.008
			95% CI	[-0.041;0.038]	95% CI	[-0.028;0.063]	95% CI	[-0.082;0.028]	95% CI	[-0.021;0.009]
			*P* value	0.948	*P* value	0.453	*P* value	0.335	*P* value	0.418

*P* values were adjusted for age and BMI. *P* values < 0.05 were shown in bold. Log transformed (log_10_) values were used for HOMA-IR, HOMA-B, Stumvoll-First phase and second phase insulin secretion. The effect allele is the allele to which the β estimate refers.

As shown in Tables 5 and 6, in the females, *ABCG2* rs2231142 was associated with fasting plasma glucose (*p* = 0.002; empirical *p* = 0.027), while *SLC17A1* rs1183210 was significantly associated with 2-h plasma glucose (*p* = 0.047; empirical *p* = 0.510). *LRP2* rs2544390 and rs1333049 showed significant correlation with fasting insulin, HOMA-IR and Stumvoll second phase insulin secretion (*p* = 0.028, 0.033 and 0.052; empirical *p* = 0.346, 0.389, 0.540; *p* = 0.034, 0.047 and 0.038; empirical *p* = 0.400, 0.493, 0.430). Meanwhile, *SLC2A9* rs11722228 was correlated with 2-h glucose, 2-h insulin and Stumvoll first phase insulin secretion (*p* = 0.024, 0.049 and 0.049; empirical *p* = 0.300, 0.511, 0.515). The uric acid-raising allele of *SLC2A9* rs16890979 was associated with lower Stumvoll second-phase index of insulin secretion (*p* = 0.039; empirical *p* = 0.441). However, the SNPs from *PDZK1, SLC17A3* and *SLC2A9-WDR* showed no association with any quantitative trait of glucose metabolism and insulin secretion in our samples.

**Table 5:** Association between SNPs from fifteen loci and glucose and insulin levels in females with normal glucose regulation.

Loci	SNP	Effect/Other Allele	Fasting glucose (n = 1375)		2-h glucose (n = 1375)		Fasting insulin (n = 715)		2-h insulin (n = 715)	
*PDZK1*	rs12129861	A/G	Beta	-0.010±0.025	Beta	-0.029±0.051	Beta	0.003±0.018	Beta	-0.013±0.024
			95% CI	[-0.060;0.039]	95% CI	[-0.130;0.071]	95% CI	[-0.033;0.038]	95% CI	[-0.061;0.034]
			*P* value	0.678	*P* value	0.568	*P* value	0.880	*P* value	0.581
*GCKR*	rs780094	G/A	Beta	-0.011±0.019	Beta	0.046±0.040	Beta	-0.009±0.014	Beta	-0.014±0.019
			95% CI	[-0.049;0.026]	95% CI	[-0.032;0.125]	95% CI	[-0.036;0.019]	95% CI	[-0.05;0.023]
			*P* value	0.553	*P* value	0.245	*P* value	0.527	*P* value	0.465
*LRP2*	rs2544390	T/C	Beta	0.007±0.019	Beta	-0.051±0.039	Beta	0.030±0.014	Beta	-0.005±0.019
			95% CI	[-0.030;0.045]	95% CI	[-0.128;0.026]	95% CI	[0.003;0.058]	95% CI	[-0.041;0.032]
			*P* value	0.706	*P* value	0.193	*P* value	**0.028**	*P* value	0.803
*SLC2A9*	rs11722228	T/C	Beta	-0.0004±0.022	Beta	-0.101±0.045	Beta	-0.022±0.016	Beta	-0.042±0.021
			95% CI	[-0.043;0.042]	95% CI	[-0.188;-0.013]	95% CI	[-0.053;0.010]	95% CI	[-0.083;0]
			*P* value	0.985	*P* value	**0.024**	*P* value	0.174	*P* value	**0.049**
*SLC2A9*	rs16890979	T/C	Beta	-0.105±0.076	Beta	-0.029±0.156	Beta	0.043±0.051	Beta	0.103±0.068
			95% CI	[-0.255;0.044]	95% CI	[-0.335;0.278]	95% CI	[-0.057;0.143]	95% CI	[-0.031;0.238]
			*P* value	0.167	*P* value	0.854	*P* value	0.397	*P* value	0.131
*SLC2A9*	rs3775948	C/G	Beta	-0.017±0.020	Beta	0.043±0.041	Beta	0.008±0.014	Beta	0.020±0.019
			95% CI	[-0.056;0.022]	95% CI	[-0.038;0.123]	95% CI	[-0.020;0.037]	95% CI	[-0.018;0.058]
			*P* value	0.398	*P* value	0.300	*P* value	0.559	*P* value	0.300
*SLC2A9-WDR1*	rs10489070	C/G	Beta	0.028±0.028	Beta	-0.064±0.059	Beta	0.016±0.021	Beta	0.012±0.028
			95% CI	[-0.028;0.084]	95% CI	[-0.178;0.051]	95% CI	[-0.024;0.056]	95% CI	[-0.042;0.066]
			*P* value	0.325	*P* value	0.278	*P* value	0.437	*P* value	0.667
*ABCG2*	rs2231142	A/C	Beta	-0.067±0.021	Beta	-0.051±0.044	Beta	0.009±0.016	Beta	0.009±0.021
			95% CI	[-0.109;-0.025]	95% CI	[-0.137;0.035]	95% CI	[-0.021;0.040]	95% CI	[-0.032;0.050]
			*P* value	**0.002**	*P* value	0.246	*P* value	0.553	*P* value	0.664
*LRRC16A*	rs742132	C/T	Beta	0.041±0.023	Beta	0.034±0.047	Beta	0.016±0.017	Beta	0.029±0.022
			95% CI	[-0.004;0.086]	95% CI	[-0.058;0.126]	95% CI	[-0.017;0.048]	95% CI	[-0.014;0.073]
			*P* value	0.073	*P* value	0.472	*P* value	0.348	*P* value	0.187
*SLC17A1*	rs1183201	T/A	Beta	-0.013±0.027	Beta	-0.109±0.055	Beta	-0.014±0.020	Beta	0.013±0.026
			95% CI	[-0.066;0.039]	95% CI	[-0.216;-0.001]	95% CI	[-0.052;0.025]	95% CI	[-0.038;0.065]
			*P* value	0.616	*P* value	**0.047**	*P* value	0.485	*P* value	0.616
*SLC17A3*	rs1165205	A/T	Beta	-0.024±0.027	Beta	-0.101±0.055	Beta	-0.015±0.019	Beta	0.004±0.026
			95% CI	[0.076;0.028]	95% CI	[-0.208;0.006]	95% CI	[-0.053;0.023]	95% CI	[-0.047;0.055]
			*P* value	0.369	*P* value	0.065	*P* value	0.438	*P* value	0.890
	rs1333049	C/G	Beta	-0.016±0.020	Beta	0.012±0.041	Beta	0.031±0.015	Beta	0.032±0.020
			95% CI	[-0.055;0.023]	95% CI	[-0.069;0.092]	95% CI	[0.002;0.060]	95% CI	[-0.006;0.071]
			*P* value	0.430	*P* value	0.777	*P* value	**0.034**	*P* value	0.102
*SLC22A11*	rs17300741	G/A	Beta	-0.069±0.043	Beta	-0.015±0.088	Beta	0.028±0.033	Beta	0.013±0.044
			95% CI	[-0.153;0.014]	95% CI	[-0.187;0.157]	95% CI	[-0.036;0.093]	95% CI	[-0.073;0.100]
			*P* value	0.106	*P* value	0.864	*P* value	0.388	*P* value	0.764
*SLC22A12*	rs506338	C/T	Beta	-0.002±0.022	Beta	-0.068±0.044	Beta	-0.009±0.015	Beta	-0.029±0.020
			95% CI	[-0.045;0.040]	95% CI	[-0.155;0.019]	95% CI	[-0.039;0.021]	95% CI	[-0.069;0.011]
			*P* value	0.916	*P* value	0.126	*P* value	0.545	*P* value	0.153
*SF1*	rs606458	G/A	Beta	-0.026±0.020	Beta	-0.014±0.042	Beta	-0.008±0.015	Beta	-0.008±0.020
			95% CI	[-0.066;0.014]	95% CI	[-0.096;0.068]	95% CI	[-0.037;0.020]	95% CI	[-0.046;0.031]
			*P* value	0.197	*P* value	0.738	*P* value	0.566	*P* value	0.700

*P* values were adjusted for age and BMI. *P* values < 0.05 were shown in bold. Log transformed (log_10_) values were used for fasting and 2-h insulin levels. The effect allele is the allele to which the β estimate refers.

**Table 6:** Association between SNPs from fifteen loci and insulin secretion and sensitivity indices in females with normal glucose regulation.

Loci	SNP	Effect/Other Allele	HOMA-IR (n = 715)		HOMA-B (n = 715)		Stumvoll-First phase (n = 715)		Stumvoll-second phase (n = 715)	
*PDZK1*	rs12129861	A/G	Beta	0.003±0.019	Beta	-0.003±0.021	Beta	-0.013±0.024	Beta	0.005;0.008
			95% CI	[-0.034;0.039]	95% CI	[-0.044;0.038]	95% CI	[-0.061;0.034]	95% CI	[-0.011;0.021]
			*P* value	0.886	*P* value	0.891	*P* value	0.581	*P* value	0.575
*GCKR*	rs780094	G/A	Beta	-0.008±0.014	Beta	-0.003±0.016	Beta	-0.014±0.019	Beta	-0.001;0.006
			95% CI	[-0.036;0.020]	95% CI	[-0.035;0.029]	95% CI	[-0.05;0.023]	95% CI	[-0.014;0.011]
			*P* value	0.580	*P* value	0.866	*P* value	0.465	*P* value	0.863
*LRP2*	rs2544390	T/C	Beta	0.031±0.014	Beta	0.028±0.016	Beta	-0.005±0.019	Beta	0.012;0.006
			95% CI	[0.002;0.059]	95% CI	[-0.004;0.060]	95% CI	[-0.041;0.032]	95% CI	[0;0.025]
			*P* value	**0.033**	*P* value	0.083	*P* value	0.803	*P* value	**0.052**
*SLC2A9*	rs11722228	T/C	Beta	-0.024±0.014	Beta	-0.014±0.018	Beta	-0.042±0.021	Beta	-0.003;0.007
			95% CI	[-0.056;0.008]	95% CI	[-0.051;0.022]	95% CI	[-0.083;0]	95% CI	[-0.017;0.011]
			*P* value	0.149	*P* value	0.436	*P* value	**0.049**	*P* value	0.647
*SLC2A9*	rs16890979	T/C	Beta	0.034±0.053	Beta	0.086±0.059	Beta	0.103±0.068	Beta	0.048;0.023
			95% CI	[-0.070;0.137]	95% CI	[-0.030;0.203]	95% CI	[-0.031;0.238]	95% CI	[0.002;0.093]
			*P* value	0.523	*P* value	0.147	*P* value	0.131	*P* value	**0.039**
*SLC2A9*	rs3775948	C/G	Beta	0.007±0.015	Beta	0.015±0.017	Beta	0.020±0.019	Beta	0.002;0.007
			95% CI	[-0.023;0.036]	95% CI	[-0.018;0.048]	95% CI	[-0.018;0.058]	95% CI	[-0.011;0.015]
			*P* value	0.657	*P* value	0.379	*P* value	0.300	*P* value	0.756
*SLC2A9-WDR1*	rs10489070	C/G	Beta	0.017±0.021	Beta	0.012±0.024	Beta	0.012±0.028	Beta	0.011;0.009
			95% CI	[-0.025;0.059]	95% CI	[-0.035;0.059]	95% CI	[-0.042;0.066]	95% CI	[-0.008;0.029]
			*P* value	0.420	*P* value	0.623	*P* value	0.667	*P* value	0.247
*ABCG2*	rs2231142	A/C	Beta	0.005±0.016	Beta	0.024±0.018	Beta	0.009±0.021	Beta	0.005;0.007
			95% CI	[-0.026;0.037]	95% CI	[-0.012;0.060]	95% CI	[-0.032;0.050]	95% CI	[-0.009;0.019]
			*P* value	0.743	*P* value	0.193	*P* value	0.664	*P* value	0.454
*LRRC16A*	rs742132	C/T	Beta	0.019±0.017	Beta	0.006±0.019	Beta	0.029±0.022	Beta	0.007;0.008
			95% CI	[-0.015;0.052]	95% CI	[-0.032;0.043]	95% CI	[-0.014;0.073]	95% CI	[-0.008;0.022]
			*P* value	0.276	*P* value	0.773	*P* value	0.187	*P* value	0.345
*SLC17A1*	rs1183201	T/A	Beta	-0.013±0.020	Beta	-0.013±0.023	Beta	0.013±0.026	Beta	0.003;0.009
			95% CI	[-0.053;0.027]	95% CI	[-0.058;0.032]	95% CI	[-0.038;0.065]	95% CI	[-0.014;0.021]
			*P* value	0.522	*P* value	0.571	*P* value	0.616	*P* value	0.725
*SLC17A3*	rs1165205	A/T	Beta	-0.015±0.020	Beta	-0.011±0.023	Beta	0.004±0.026	Beta	0.001;0.009
			95% CI	[-0.054;0.024]	95% CI	[-0.055;0.033]	95% CI	[-0.047;0.055]	95% CI	[-0.016;0.018]
			*P* value	0.459	*P* value	0.619	*P* value	0.890	*P* value	0.918
	rs1333049	C/G	Beta	0.030±0.015	Beta	0.031±0.017	Beta	0.032±0.020	Beta	0.014;0.007
			95% CI	[0;0.060]	95% CI	[-0.002;0.065]	95% CI	[-0.006;0.071]	95% CI	[0.001;0.027]
			*P* value	**0.047**	*P* value	0.066	*P* value	0.102	*P* value	**0.038**
*SLC22A11*	rs17300741	G/A	Beta	0.016±0.034	Beta	0.062±0.038	Beta	0.013±0.044	Beta	0.012;0.015
			95% CI	[-0.050;0.082]	95% CI	[-0.013;0.137]	95% CI	[-0.073;0.100]	95% CI	[-0.017;0.041]
			*P* value	0.636	*P* value	0.106	*P* value	0.764	*P* value	0.420
*SLC22A12*	rs506338	C/T	Beta	-0.008±0.016	Beta	-0.014±0.018	Beta	-0.029±0.020	Beta	-0.002;0.007
			95% CI	[-0.039;0.023]	95% CI	[-0.049;0.020]	95% CI	[-0.069;0.011]	95% CI	[-0.016;0.011]
			*P* value	0.615	*P* value	0.419	*P* value	0.153	*P* value	0.740
*SF1*	rs606458	G/A	Beta	-0.010±0.015	Beta	-0.005±0.017	Beta	-0.008±0.020	Beta	-0.003;0.007
			95% CI	[-0.039;0.020]	95% CI	[-0.038;0.029]	95% CI	[-0.046;0.031]	95% CI	[-0.016;0.010]
			*P* value	0.528	*P* value	0.786	*P* value	0.700	*P* value	0.655

*P* values were adjusted for age and BMI. *P* values < 0.05 were shown in bold. Log transformed (log_10_) values were used for HOMA-IR, HOMA-B, Stumvoll-First phase and second phase insulin secretion. The effect allele is the allele to which the β estimate refers.

## Discussion

In this study, we attempted to investigate the effects of recently reported uric acid-associated loci on the risk of T2D as well as the quantitative traits related to glucose metabolism and insulin secretion in a Chinese population. We confirmed the association of *GCKR* and *SF1* with T2D. Additionally, in the males, we observed that *LRRC16A* rs742132 were associated with the serum 2-h insulin and insulin secretion indices; whereas *SLC22A11* rs17300741 had an impact on β-cell function and insulin secretion. The SNP of rs506338 in *SLC22A12* showed strong influence on β-cell function as well as *SLC2A9* rs3775948 significantly affected the serum 2-h glucose. In the females, the uric acid-raising alleles of *LRP2* rs2544390 and rs1333049 were associated with an increased fasting insulin level, HOMA-IR and second phase insulin secretion. The uric acid-raising T allele in *SLC2A9* rs11722228 could not increase the level of 2-h glucose; however, it elevated the 2-h insulin and first phase insulin secretion. The uric acid-lowering alleles of *SLC2A9* rs16890979 and *SLC17A1* rs1183210 were associated with increased second phase insulin secretion and the 2-h glucose levels, respectively. The risk allele of elevated uric acid levels in *ABCG2* rs2231142 was shown to increase the fasting glucose levels. However, the effect of these SNPs on glucose metabolism and insulin secretion (i.e., an increased or decreased effect) was not consistent. This finding suggests that exploring the potential mechanisms of these loci on glucose metabolism and insulin secretion is important in elucidating the links between uric acid disorders and T2D.

We demonstrated that the SNPs from *GCKR* and *SF1* had an effect on T2D in our samples. Note that *GCKR* was reported to be independently susceptible to T2D in multiple populations [21, 22]. Recently, *GCKR* involvement was identified in regulating serum uric acid levels [19, 23, 24]. In addition, the uric acid-raising allele in *GCKR* [14] could increase the risk of T2D in the Chinese population. This result further highlights the link between uric acid disorders and T2D. *SF1* rs606458 has been associated with uric acid levels in individuals of African American, European and Chinese ancestry. In accordance with the effects of *GCKR*, the uric acid-raising allele in *SF1* (my paper) could increase the risk of T2D in a Chinese population. However, as the cultural diversity, risk allele frequency and pattern of linkage disequilibrium differed in various populations, reports on the risk allele of uric acid in *GCKR* and *SFI* from foreign studies contradicted that in the Chinese population [23–25]. We failed to detect the effects of SF1 on T2D in males, and previous studies have demonstrated the effects of SF1 on uric acid levels in males. Based on these inconsistencies, further study is needed.

In the males, the uric acid-raising A-allele in *LRRC16A* rs742132 were associated with the increase of serum 2-h insulin and insulin secretion. *LRRC16A* rs742132 was reported to exert strong effects on serum uric acid concentration and gout, but the functional role of this SNP remains unclear. Combined with the results that the rs742132 played roles in insulin secretion, further studies are necessary to investigate whether this intronic SNP would regulate *LRRC16A* gene expression when involved in the uric acid and insulin metabolism. In addition, the G-allele in *SLC22A11* rs17300741 causes lower uric acid levels, improved β-cell function and elevated insulin secretion. Human organic anion transporter 4 (OAT4/SLC22A11) is expressed on the apical membrane of renal proximaltubule cells and placenta in the kidney and mediates the transport of uric acid [26–28]. The uric acid-raising G-allele in *SLC22A11* rs17300741 plays roles similar to those of the uric acid-lowering A-allele in *LRRC16A* rs742132. The strength of the associations between the *SLC22A11* and *LRRC16A* loci was not affected by the serum uric acid levels or other confounders, which suggests a greater likelihood of a direct effect on insulin secretion and insulin resistance.

In the females, the uric acid-raised allele in *LRP2* rs2544390 and rs1333049 was significantly correlated with increased fasting insulin levels, HOMA-IR and Stumvoll second phase insulin secretion. *LRP2* encodes low-density lipoprotein-related protein 2, which is a member of the low-density lipoprotein receptor gene family. Few studies have reported its association with uric acid transportation. *LRP2* is a multi-ligand receptor expressed in various tissues, predominantly in the kidneys, particularly in the glomeruli and proximal tubular cells; therefore, *LRP2* may play a role in renal reabsorption through its ligands, including insulin [29, 30]. This study showed that *LRP2* increased serum insulin levels and insulin secretion and reduced insulin sensitivity, which could, conversely, affect *LRP2*-ligands and result in elevated uric acid levels through enhanced renal reabsorption. *SLC2A9* rs11722228 was correlated with 2-h glucose, 2-h insulin and Stumvoll first phase insulin secretion. SLC2A9, as a glucose and uric acid transporter facilitator, could deregulate the glucose-stimulated insulin secretion in pancreatic β-cells [31, 32]. In the first phase of insulin secretion, β-cells sense extracellular glucose concentration through the uptake of glucose by the glucose transporter. According to recent reports, GLUT9 is expressed in β-cells and is expected to participate in glucose sensing in β-cells [31], which may elucidate the finding that the uric acid-raising allele *SLC2A9* rs11722228 simultaneously increased 2-h glucose and insulin secretion. The uric acid-raising allele of *SLC2A9* rs16890979 was associated with decreased second phase insulin secretion, which may be attributed to the polygenetic background of uric acid disorders and T2D and associated complex environmental factors. Note that elevated uric acid levels and insulin resistance interacted with each other. Elevated uric acid levels lead to insulin resistance through two mechanisms. First, the endothelial dysfunction caused by increased uric acid levels may reduce the endothelial NO production, which plays a role in insulin action [33]. The reduced endothelial NO levels could lower blood flow to the skeletal muscle and peripheral tissues and decrease glucose uptake, which may lead to or aggravate insulin resistance [34]. Second, uric acid induces inflammation and oxidative stress in cultured adipocytes [35]. While in the adipocyte, inflammation and oxidative stress could contribute to insulin resistance [36]. Alternatively, insulin resistance could result in hyperinsulinemia; thus, high levels of insulin could stimulate the proximal tubule brush border in the kidneys, promote the exchange of uric acid and sodium ions, increase uric acid reabsorption, reduce uric acid clearance and elevate uric acid levels [37]. In addition, as a member of the ATP-binding cassette superfamily of membrane transporters, *ABCG2* rs2231142 also act as a uric acid transporter and it is well-known that the dysfunction of ABCG2 can cause hyperuricemia [38]. The present study showed strong association between this SNP with fasting glucose. The disorder of serum uric acid and glucose levels is regulated by many complex factors in humans, including genetic contribution. Nowadays, few study is conducted to discover the genetic links between the rs2231142 and glucose. With the identification of the uric acid-raising allele elevating glucose levels, the functional experiments is needed to further explore the role of ABCG2 in regulating glucose levels.

This study has some limitations. First, we cannot exclude the possibility of a false positive in our findings when analysed multiple traits and SNPs in this study. As these 14 SNPs were originally identified and confirmed by many large-scale studies to be associated with uric acid as well as all traits were highly related, the effect of multiple comparisons may be limited. Second, we only analysed the effects of these uric acid loci on glucose metabolism and insulin secretion in the normal glucose regulation subjects because most of the type 2 diabetes patients were receiving glucose lowering therapy. However, according to recent reports, the effect of genetic variation on insulin secretion depends on glycaemia [38, 39], future investigation is needed to explore the effects of these variants on glucose metabolism and insulin secretion. Third, only the reported uric acid-associated loci were analysed in this study. Because uric acid disorders were significantly associated with T2D, glucose metabolism and insulin secretion, a future large-scale study is needed to explore in detail the potential genetic links between these factors. Fourth, as rs16890979 and rs17300741 are rare in Chinese population, the present study had limited power to detect the effect of these SNPs on T2D, glucose metabolism and insulin secretion. Finally, we didn’t adjust the other confounding factors, such as diet, alcohol consumption and cigarette smoking, as these may bias the association between uric acid-associated genes and T2D, glucose metabolism and insulin secretion.

We analysed the effects of the SNPs from eleven uric acid-associated loci on the risk of T2D, glucose metabolism and insulin secretion and showed that *GCKR* rs780094 and *SF1* rs606458 variants have an impact on the risk of T2D and *LRRC16A, SLC22A11* and *SLC22A12* play roles in regulating glucose metabolism and insulin secretion in Chinese males as well as *ABCG2, SLC17A1* and *LRP2* in Chinese females. The variants in *SLC2A9* modulate glucose metabolism and insulin secretion in both Chinese males and females. Future large-scale studies should further investigate the potential genetic links between uric acid-associated loci and glucose metabolism and insulin secretion in multiple populations.
